# Detection and imaging of gadolinium accumulation in human bone tissue by micro- and submicro-XRF

**DOI:** 10.1038/s41598-020-63325-9

**Published:** 2020-04-14

**Authors:** Anna Turyanskaya, Mirjam Rauwolf, Vanessa Pichler, Rolf Simon, Manfred Burghammer, Oliver J. L. Fox, Kawal Sawhney, Jochen G. Hofstaetter, Andreas Roschger, Paul Roschger, Peter Wobrauschek, Christina Streli

**Affiliations:** 1 TU Wien, Atominstitut, Stadionallee 2, Vienna, 1020 Austria; 2 Karlsruher Institute for Technology (KIT), ANKA Synchrotron Radiation Source, Hermann-von-Helmholtz-Platz 1, Eggenstein-Leopoldshafen, Karlsruhe, 76344 Germany; 3 European Synchrotron Radiation Facility, Avenue des Martyrs 71, Grenoble, 38043 France; 4 Diamond Light Source Ltd, Harwell Science and Innovation Campus, Didcot, Oxfordshire OX11 0DE UK; 5 Ludwig Boltzmann Institute of Osteology at the Hanusch Hospital of WGKK and AUVA Trauma Centre Meidling, 1st Medical Department Hanusch Hospital, Heinrich-Collin-Strasse 30, Vienna, 1140 Austria; 6 Orthopaedic Hospital Vienna-Speising, Speisinger Strasse 109, Vienna, 1130 Austria; 7 Paris-Lodron-University of Salzburg, Department of Chemistry and Physics of Materials, Jakob-Haringer-Strasse 2a, Salzburg, 5020 Austria

**Keywords:** Imaging, Bone, Imaging techniques

## Abstract

Gadolinium-based contrast agents (GBCAs) are frequently used in patients undergoing magnetic resonance imaging. In GBCAs gadolinium (Gd) is present in a bound chelated form. Gadolinium is a rare-earth element, which is normally not present in human body. Though the blood elimination half-life of contrast agents is about 90 minutes, recent studies demonstrated that some tissues retain gadolinium, which might further pose a health threat due to toxic effects of free gadolinium. It is known that the bone tissue can serve as a gadolinium depot, but so far only bulk measurements were performed. Here we present a summary of experiments in which for the first time we mapped gadolinium in bone biopsy from a male patient with idiopathic osteoporosis (without indication of renal impairment), who received MRI 8 months prior to biopsy. In our studies performed by means of synchrotron radiation induced micro- and submicro-X-ray fluorescence spectroscopy (SR-XRF), gadolinium was detected in human cortical bone tissue. The distribution of gadolinium displays a specific accumulation pattern. Correlation of elemental maps obtained at ANKA synchrotron with qBEI images (quantitative backscattered electron imaging) allowed assignment of Gd structures to the histological bone structures. Follow-up beamtimes at ESRF and Diamond Light Source using submicro-SR-XRF allowed resolving thin Gd structures in cortical bone, as well as correlating them with calcium and zinc.

## Introduction

The method of contrast-enhanced MRI (CE-MRI) dates to 1988, when the first gadolinium (Gd)-based contrast agent (GBCA), gadopentetate dimeglumine (Magnevist, Bayer), was approved for clinical use. CE-MRI is a useful imaging tool nowadays, with approximately 30 million procedures done each year worldwide, and more than 300 million procedures already performed to date^1^. Estimates show that the GBCAs are administered in 25–35% of all MRI examinations^1,2^.

According to the data available, most of the administered dose of GBCAs (regardless of an agent used), should be cleared in less than 2 and >95% by 24 hours^3^. Recent findings, however, demonstrate that the GBCAs are not fully excreted and the accumulation of Gd in, e.g. neuronal tissues and organs such as brain takes place^4^.

The GBCAs are considered to be rather safe, yet there is a number of papers reporting nephrotoxic, hematotoxic, hepatotoxic and neurotoxic effects observed in animals and humans^5^. One of the most serious adverse reactions associated with the use of GBCAs is nephrogenic systemic fibrosis (NSF), which had been observed in patients with renal insufficiency^6^. NSF is a rare, but serious disease characterized by widespread tissue hardening (primarily skin is affected) with fibrotic nodules and plaques. First described in 1997, it was finally linked to the use of GBCAs in patients with kidney dysfunction in 2006^6,7^. The pathophysiological mechanism involved remains unclear, though the dissociation of Gd from GBCA is usually indicated as the causative factor^3,8^. Due to renal insufficiency, the elimination of GBCA will be reduced, more of the dissociated Gd will be available, leading to tissue accumulation and further manifestation of Gd toxicity. The linear GBCAs due to their lower thermodynamic and kinetic stability (compared to macrocyclic GBCAs) are now considered as high-risk agents in patients with severe renal disease^9^.

The analysis by secondary ion mass spectrometry (SIMS) and inductively coupled plasma mass spectrometry (ICP-MS) techniques of skin biopsies from patients suffering from NSF after GBCA intake revealed Gd accumulations, which were inhomogeneous and co-localized with calcium^10^ and phosphorus^11^. In 2010 application of synchrotron radiation induced X-ray fluorescence spectroscopy (SR-XRF) for investigation of skin samples of NSF patients independently by High *et al*. and George *et al*. also confirmed depositions of Gd coinciding with other elements^12,13^. Multiorgan Gd depositions in NSF patient, involving skin, liver, lungs, intestinal wall (ileum), kidney, lymph node, skeletal muscle, dura mater and cerebellum, were reported by Sanyal *et al*. by performing scanning electron microscopy (SEM) with energy-dispersive X-ray spectroscopy (EDS) analysis^14^.

There is a growing body of research devoted to the investigation of possible GBCAs adverse effects in patients with normal renal function and, specifically, the retention of Gd. It would not be an overstatement to say, that a main focus lies on the retention of Gd by brain tissues. A recent study by Flood *et al*. connects increased signal intensity in children with repeated exposure to linear GBCAs^15^. Again, the use of less stable linear GBCAs is more likely to result in the deposition, however studies demonstrate, that the macrocyclic agents might be retained as well^4,16^. In view of this, the European Medicines Agency (EMA) issued recommendations for the restriction of linear GBCAs in body scans^9^.

Recently, accumulation of Gd in bone tissue received deserved attention, especially given the fact that bone tissue retains Gd from GBCAs to a greater degree (23 times higher levels) compared to brain^16^. Darrah *et al*. used ICP-MS to measure Gd content (bulk) in bone tissue in osteoporotic and osteoarthritic (previously exposed to Gd) and osteoarthritic (non-exposed, used as controls) patients, and concluded that Gd can be retained for longer than 8 years after the exposure^17^. Series of *in vivo* experiments by M. Lord *et al*. contributed substantially to the current knowledge about Gd retention. The group developed an *in vivo* XRF device and obtained quantitative data on Gd content in the bone tissue of exposed patients in comparison with control subjects^18,19^.

However, very little is known about the distribution of Gd in bone on the microscopic level. Our group has already employed the method of synchrotron radiation induced micro-X-ray fluorescence (SR-micro-XRF) for the characterization of distribution of trace elements, such as lead (Pb), zinc (Zn) and strontium (Sr) in healthy and diseased bone and cartilage samples^20–22^. Within this project we applied SR-micro- and submicro-XRF for the detection and mapping of Gd distribution within bone tissue. To the best of our knowledge these measurements are the first attempt to image Gd accumulations in the bone.

## Materials and methods

### Patient and bone samples preparation

Patient - male individual, 54 y, diagnosed with male idiopathic osteoporosis, multiple vertebral fractures and low bone mineral density, without indication of renal impairment. A transiliac crest biopsy was harvested from the patient for diagnostic reasons. All study-related procedures were approved and supervised by the local ethical committee of the St. Vincent Hospital Vienna, Austria, an academic teaching hospital of the Medical University of Vienna following the Declaration of Helsinki (DoH). The patient provided written consent for anonymized outcomes to be further used in scientific examinations.

It is known that the patient received MRI eight months prior to biopsy. Unfortunately, up to date we were not able to confirm whether and which contrast agent was used. No other sources of Gd intake are known.

The biopsy was dehydrated and fixed in ethanol and embedded in polymethylmethacrylate (PMMA). In order to avoid contaminations, only fresh and clean solutions of pro analysis quality were used during sample preparation. Typical contamination originating from the polishing procedure and consisting of Cr-Mn-Fe-Ni was identified in only one sample area and routinely excluded in the data analysis (sample demonstrated in supplementary material). The thick PMMA block was trimmed using a low-speed diamond saw (Buehler Isomet, Lake Pluff, USA), and further sandpapered with decreasing grit size and finally polished by silk cloths loaded with diamond grains (3 μm and 1 μm) using a precision polishing device (PM5; Logitech Ltd., Glasgow, UK). This ensured the block having a smooth and flat surface, which is necessary for both quantitative backscattered electron imaging (qBEI) and SR-micro-XRF measurements (ANKA).

A 3 µm-thin cut was prepared out of the PMMA block for the measurements on two high-resolution SR-submicro-XRF beamlines (ESRF and Diamond Light Source synchrotrons), using a microtome (LEICA SM2500; Leica Microsystems GmbH, Wetzlar, Germany). The cutting was performed as close as possible to the original surface – we estimate the offset to less than 15 µm. The resultant cut was sandwiched between two 8 µm Kapton foils and fastened to a plexiglass frame.

### Quantitative backscattered electron imaging (qBEI)

qBEI was used to determine the degree of tissue mineralization. Making use of the dependency of the backscatter electron coefficient on the local electron density (average atomic number) of the target material, this method allows to derive the degree of mineralization of bone and further to evaluate the calcium concentration (weight percent, wt% Ca). Hence, bright areas in the grey scale qBEI images refer to higher, and the dark ones to lower mineralized matrix (higher vs. lower Ca content). This method requires flat polished surface in order to derive quantitative information, therefore is only possible for bone samples as PMMA embedded blocks. The measurements were preformed using a digital scanning electron microscope equipped with a four quadrant semiconductor backscattered electron detector (DSM 962; Zeiss, Oberkochen, Germany), operated at 20 keV beam energy. Areas were scanned with ×200 nominal magnification (pixel resolution 1 μm). The information depth of qBEI depends on the mineralization and is in the range of 1–1.5 μm. The details on the technique can be found in ref. ^23^

### SR-XRF measurements and data processing

#### ANKA

**Figure 1:**
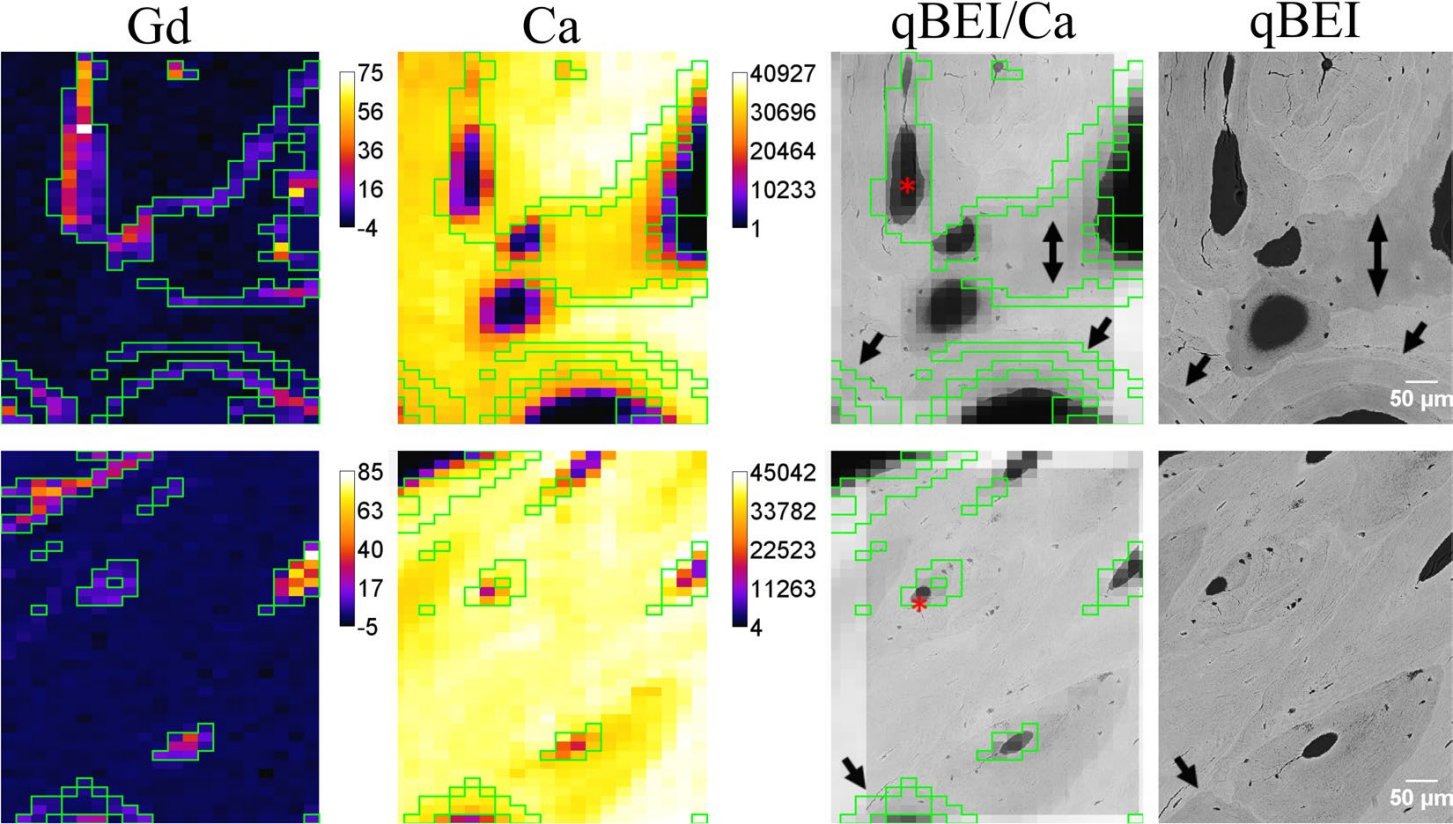
Elemental maps of Gd and Ca (in cps) obtained at ANKA, composite qBEI/Ca overlay and corresponding qBEIs of two areas. The area size for maps of 21 × 41 pixels corresponds to 500 × 600 µm^2^; for qBEIs 450 × 570 µm^2^. The black arrows (composite image and qBEI) point at cement lines; red asterisk – canals.

The measurements on the PMMA block sample were performed at the confocal FLUO beamline at ANKA (KIT, Karlsruhe, Germany). For the first beamtime (two areas on the surface of the block, Fig. 1) beam size was defined at Ti-Ka (4.51 keV) as 27 μm × 18 μm × 24.5 µm (hor. × vert. × dep.), with the excitation energy of 9.2 keV (the energy optimized for the detection of manganese due to the original purpose of the experiment). The step size was 25 µm × 15 µm. For the second beamtime (another two areas, supplementary material Fig. S1) the beam size was defined at Ti-Ka as 28 μm × 19.5 μm × 35 µm (hor. × vert. × dep.), the step size was 25 µm × 17 µm, same excitation energy 9.2 keV. In both measurement sessions the setup included W-Si multilayer monochromator, 2 polycapillary half-lenses, 20 µm Al detector-filter, Vortex 50 mm^2^ silicon-drift detector, and XIA Mercury digital pulse processor. The angle between source and detector was 90°. The acquisition time was 15–17 seconds per spot (pixel) depending on the area.

#### ESRF

Measurements on the thin section with submicrometer resolution were performed at the ID13 microfocus beamline at ESRF (Grenoble, France); beam size 50 nm, defined using Siemens star standard. The excitation energy was set to 12.7 keV (corresponds to highest flux available at the beamline). The beamline was equipped with multilayer Laue lens optics, Vortex-EM silicon drift detector (Hitachi, USA), positioned on the rear side, “behind” the sample (i.e. the angle between source and detector is more than 90°). The step size was 500 nm with 20 s acquisition time per spot.

#### Diamond Light Source

Measurements on the same slice as analysed at the ESRF were also conducted at submicro-X-ray fluorescence setup on the B16 beamline at Diamond Light Source (Didcot, UK). The excitation energy was set to 12.7 keV to match that used at ESRF. The beam size (FWHM at 12.7 keV) was determined as about 500 nm × 610 nm (V × H) – taking into account the overlap in 45° geometry – using a 50 µm-diameter Au wire knife edge. The setup included a RuB_4_C multilayer monochromator, 150 µm pinhole and a single-element 90 Ex Vortex detector (50 mm^2^), positioned at 90° to the beam. The step size was 500 nm with an acquisition time 15 s per spot. Additional information on the setup we used for the experiment can be found in ref. ^24^

### Data processing

For the ANKA dataset, the spectra were processed using AXIL software^25^. Spectra recorded at ESRF and Diamond Light Source were processed with PyMCA^26^. The quality of fitting was further validated and text images for each element of interest were obtained using in-house software LP-map^27^. Finally, ImageJ^28^ was used to process the text images and to produce the elemental maps and overlays. Elemental maps are presented in counts per second (cps).

## Results

The SR-micro-XRF measurements in the cortical region of the human iliac crest biopsy revealed highly localized accumulations of Gd. This observation was done at the ANKA synchrotron during the beamtime dedicated to the detection of manganese. The two scans recorded during this first beamtime are presented in Fig. 1. During the follow-up beamtime we scanned two additional areas, but as the setup was adjusted differently, it was less sensitive to Gd. For illustrative purposes those two additional areas are demonstrated in supplementary material (Fig. S1).

Figure 1 shows Gd and Ca elemental maps as well as a composite image, where qBEI is superimposed on the Ca map (marked as qBEI/Ca). On the Gd map all the pixels above a certain threshold (in the range of 1–3 counts per second (cps), selected individually for each map) are selected and marked via mask (green lines). The exact same mask is applied to the Ca map and, finally, the Ca map with the mask is superimposed on the qBEI, creating the qBEI/Ca composite image. The resolution of qBEI images is higher than that of the elemental maps and facilitates assignment of the Gd structures. Indeed, it can be seen, that Gd accumulation happens within the specific restricted compartments: (i) in cement lines separating two bone packets (signified with black arrows on Fig. 1), and (ii) at the interface between mineralized matrix and vascular canals, corresponding to the walls of Haversian and Volkmann’s canals (marked with red asterisks).

The alleged cement line Gd structures are mainly 1-pixel thick (elemental maps), however the correct estimation of their thickness is not possible based on these maps only. As can be seen from the qBEI image, cement lines have a thickness <5 µm. Hence, the confocal system at ANKA with the resolution of ~20 µm can be used for locating the Gd accumulation, but gives an overestimation of the sizes of those structures, while underestimating the relative elemental content.

Therefore, as a next step, we aimed to resolve the thin structures within the mineralized bone tissue in higher resolution. These scans were performed at ESRF and Diamond Light Source synchrotrons at beamlines featuring submicrometer beam sizes. The non-confocal setups on both synchrotrons call for thin samples, therefore a slice of 3 µm thickness was prepared out of the original PMMA block as close as possible to the qBEI-measured surface (see Materials and Methods). As the excitation energy was higher compared to the ANKA synchrotron, we detected a broader range of elements including zinc (Zn), which is also known to accumulate in cement lines^22^. Therefore, correlating Gd and Zn distributions provided additional information.

**Figure 2:**
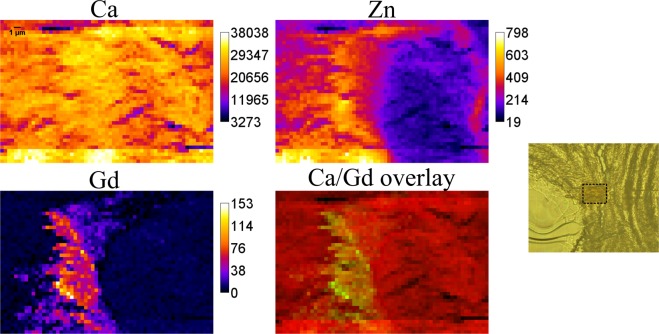
Elemental maps (in cps), composite image obtained at ESRF and light microscopy. Area size is 61 × 41 pixels corresponding to 30 × 20 µm^2^.

Figure 2 shows the elemental maps of Ca, Gd and Zn obtained at ESRF. The Ca map confirms that the entire field of view was positioned within the mineralized matrix. It can be seen, that Gd is only found in a restricted region, roughly the horizontal width of this structure can be estimated as 10–15 µm. Gd shows a unique distribution, and remarkably, it is exclusively detected in the area, where also Zn is present in high content. As reliable qBEI imaging cannot be performed on thin sections, here the composite image is prepared by overlaying Ca (red) and Gd (green) elemental maps. Due to the high intensity of the beam at ESRF, the area mapped there was subject to radiation damage and can be recognised by darker colour compared to the surrounding tissue – the respective area is marked on the light microscopy.

**Figure 3:**
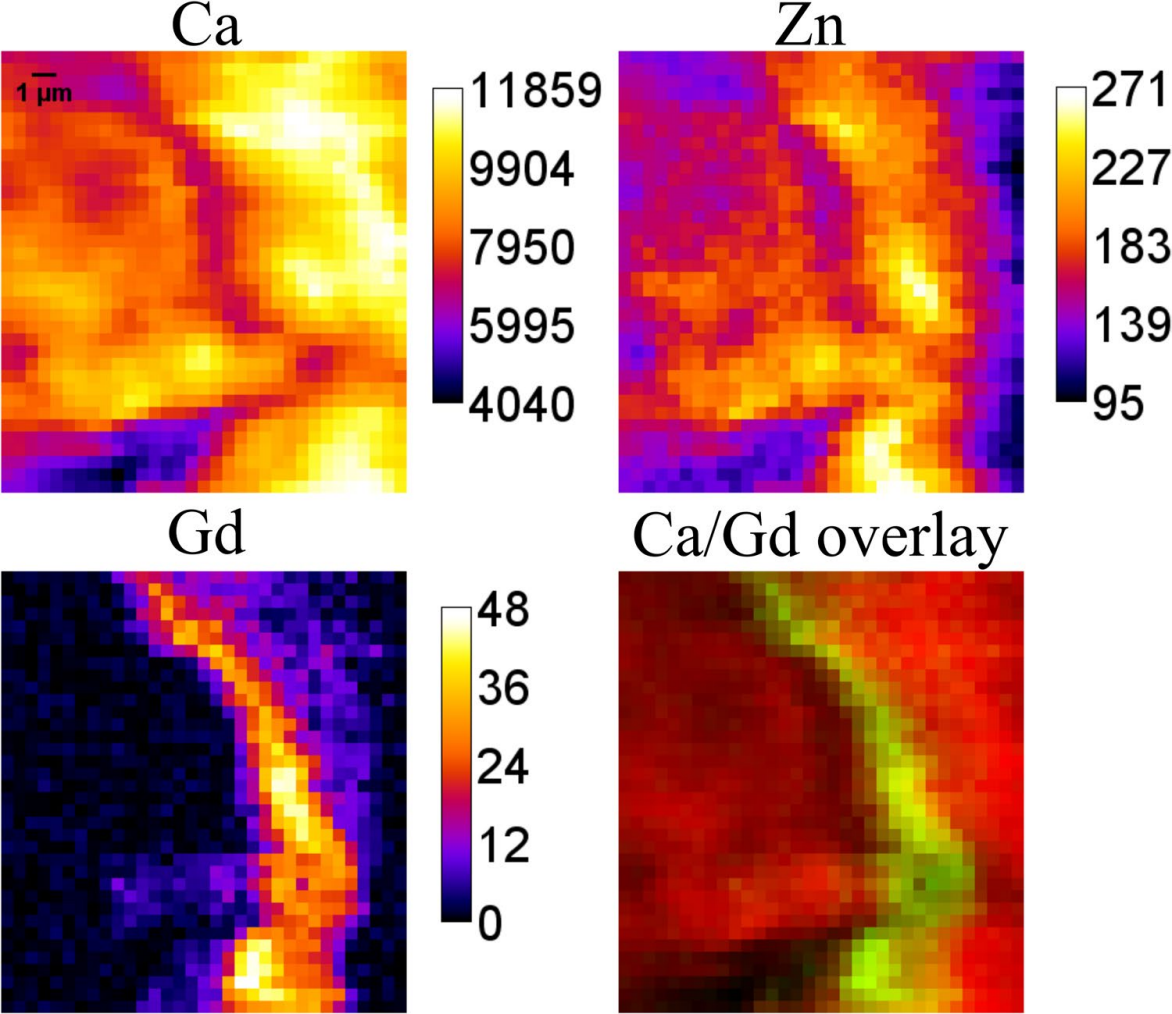
Elemental maps (in cps) and composite image obtained at Diamond Light Source. Area size is 33 × 36 pixels corresponding to 16 × 17.5 µm^2^.

The very same sample which we have measured at ESRF, although a different area of it, was later investigated during the beamtime at Diamond Light Source. Figure 3 shows the elemental maps of Ca, Gd and Zn obtained at the Diamond Light Source and the composite image of Ca (red) and Gd (green) overlay. The horizontal width of this Gd structure is around 10 µm. In this experiment Gd found to be located within the calcified area as well – and again Zn and Gd locally correlate, supporting the previous observations based on the ESRF measurements.

It should be noted that we also detected cobalt (Co) in both regions measured at ESRF and Diamond Light Source – exhibiting a similar local distribution as Ca, though its origin remains unknown.

The exemplary spectra from ANKA and ESRF synchrotrons, Co maps (ESRF and Diamond Light Source), as well as additional composite images of Zn and Gd overlays (ESRF and Diamond Light Source) are included in the supplementary material (Figs. S2, S3, and S4 respectively).

## Discussion and Conclusion

### Sources of exposure

In the present paper we demonstrated the distribution of Gd within cortical bone tissue, and its correlation with the other detected elements. It is known from the patient’s history, that MRI was carried out 8 months prior to the biopsy, but it remains unknown whether a contrast agent was used. Hence, we cannot assert that the Gd signal originates from retention from this single exposure event. It is fair to note, that other than CE-MRI sources of exposure to Gd are possible^29,30^.

Rare-earth elements (REE) mining and processing can be mentioned in this respect, although the reports on the occupational exposure associated with negative health outcomes other than pneumoconiosis are sporadic. Li *et al*. found higher urinary levels of La, Nd, Ce and Gd in exposed workers dealing with Ce and La oxides ultrafine particles and nanoparticles compared to non-exposed^31^, however the next paper from the group (presumably on the same subjects), on creatinine adjusted urinary levels of REEs, did not show significant difference in gadolinium levels^32^. We were unable to find reports on deposition of gadolinium in occupationally exposed subjects.

In the recent years the anthropogenic Gd pollution, especially in aquatic systems, became a topic of extensive research^33–35^. However, we doubt that the low concentrations of Gd in tap water could lead to the found accumulation in bone tissue; and given the likely subject’s occupational history, the likelihood of presumable occupational exposure to elevated concentrations of gadolinium is remote.

Our hypothesis is that the observations made in this study could be generally characteristic for the Gd uptake by bone, regardless of the source. Imaging experiments on larger number of biopsies from patients with known history of Gd exposure are required to systematically determine Gd accumulation/retention with respect to the source and are planned to be done after this pioneer study.

### Analysis *ex vivo*, localization within bone

To date the Gd retention in bone was investigated in bulk, and in many cases ICP-based techniques were employed for the analysis^16,17,36,37^. Research undertaken by inductively coupled plasma atomic emission spectroscopy (ICP-AES) and ICP-MS compared linear GBCA (Omniscan) to macrocyclic (ProHanse) and revealed higher levels of retention in bone in case of linear contrast agent^36,37^. One investigation employing SEM-EDS for the analysis reported that no Gd was detected in the bone. That might be due to the detection limits of the chosen method or due to the preceding sample preparation, as the sample had been decalcified^14^. While the detection limits of EDS are typically around 0.5 wt%, SR-XRF spectrometry (as employed in the present study) features a much higher sensitivity down to sub-ppm^24^, thus making this method more suitable for the local analysis of trace elements like Gd. Indeed, the anticipated Gd concentrations in bone after exposure to GBCA are within few ppm, which is supported by the bulk measurements (up to 1.77 µg Gd/g bone^37^) and *in vivo* experiments (mean 1.19 µg Gd/g bone^19^).

Since the concentration of gadolinium in the detected structures is of considerable interest, we attempted the quantification of local Gd content - and obtained the maximum values in the range of 70–270 µg/g (locally detected maximum concentration, indicative, not to be confused with bulk values mentioned above!) – the detailed procedure is described in supplementary material. This correlates well with the results obtained in a comparable elemental imaging experiment using skin biopsy, where the hot spot concentrations in Gd maps exceed 100 μg/g^38^.

By correlating elemental maps obtained at ANKA synchrotron and qBEI images, we were able to assign the histological structures, which seem to be prone to Gd accumulation, namely (i) cement lines and (ii) vascular pore walls (interface to Haversian/Volkmann’s canals). Conceivably, deposition within the walls of canals is due to the direct vicinity to the blood vessels which feature the main delivery pathway of Gd after exposure. The cement lines are marking the osteon boundaries, they are mineral-rich and collagen deficient (compared to the mineralized matrix of the osteon), and also contain non-collagenous proteins, such as osteopontin, glycosaminoglycans, osteocalcin, and bone sialoprotein^39^. The cement lines are laid down at the reversal phase of osteon formation (i.e. before the formation of the new sequential lamellae)^40^. Supposing that the transient Gd exposure occurred at this phase of osteon formation, it is plausible, that Gd may be included in the composition of the cement line and adjacent lamellas.

### Correlation with other elements, possible mechanisms of retention

The correlation of Gd with other elements can shed light on the chemical environment of Gd within the accumulations and on the mechanism of retention. Data available on Gd depositions in skin suggest a colocalization with such elements as Ca, P and Zn. Abraham *et al*. observed Gd in association with Ca by SIMS^10^. Birka *et al*. used LA-ICP-MS and concluded, that matching Gd and P distributions suggest the presence of insoluble deposits of GdPO_4_ in the tissue section; and the Gd and Ca correlation could suggest that Gd causes calcium-containing depositions, which trigger calcification^11^. George *et al*. investigated Gd accumulation in skin affected by NSF using SR-XRF, and found clear correlation between Gd, Ca and P distributions, and the use of extended absorption fine structure spectroscopy (EXAFS) further allowed to assume Gd presence in form of GdPO_4_-like structures^13^. An inhomogeneous Zn distribution was also found throughout the Gd and Ca deposits, though it was concluded that Zn does not show a simple correlation with those elements in skin. At the same time, High *et al*. also with the use of SR-XRF observed colocalization of Gd, Ca and Zn in skin tissue and hypothesized that Ca and Zn facilitate displacement of Gd from the chelating agent^12^. Interesting results were obtained by Clases *et al*., who investigated not only skin, but also brain depositions, using LA-ICP-MS. In skin elemental distribution of Gd, P, Ca and Zn correlated in location and shape, pointing to the abundance of insoluble phosphate species, while in brain correlations and co-localisation of Gd with P, Ca, Zn, as well as Fe was observed^38^.

However, despite of all the ongoing research, the mechanism of the Gd incorporation in bone remains undetermined, and the form, in which it is deposited, is not known. The investigation of spatial distribution of Gd within bone can be instrumental in understanding that, and such studies are called for^41^. Although skin deposition mechanism might differ from the accumulation in bone, we also found Gd in calcified regions. Darrah *et al*. suggested, that ionic Gd^3+^ released from Gd-chelates is subsequently incorporated into the carbonated calcium hydroxyapatite mineral phase of bone^17^. Such a process, so-called “transmetallation”, in which the molecule of GBCA supposedly undergoes in *in vivo* environment, refers to the competition between endogenous cations (Fe^3+^, Zn^2+^, Mg^2+^, Ca^2+^, etc.) and Gd^3+^, as well as between endogenous anions (carbonate, hydroxide, phosphate, etc.) and the ligand. The Ca-transmetallation is supported by Gd similarity to Ca, the ionic radii of the ions are 107.8 pm for Gd and 114 pm for calcium. In this context, we would like to mention our previous study on Sr incorporation into bone, as Sr is also chemically similar to Ca. In patients who received Sr ranelate for treatment of osteoporosis, Sr was predominantly found in the newly formed bone matrix (formed in the period of increased Sr serum levels) and it was incorporated into the hydroxyapatite crystals changing/increasing the crystal lattice constant^42,43^. Therefore, we could hypothesise, that Gd retention is of the same nature, which can be further assessed by speciation analysis.

The other possible transmetallation competitor is Zn, which was already suggested by some of the *ex vivo* measurements of skin depositions discussed above. In 2010, S. Greenberg published a case report on a patient with chronic Zn poisoning, pointing at possible Gd retention due to Gd-Zn transmetallation^44^. With our measurements performed at Diamond Light Source and ESRF synchrotrons with submicrometer beams we focused on the Gd structures within mineralized bone. These imaging experiments revealed a local overlapping of Gd and Zn. Although their distribution patterns are not the same, Gd appears to be present only in the areas of high Zn content. The previous investigations by our group demonstrated high content of Zn, Pb and Sr in the cement lines^22^. Present findings showing interdependencies between Gd and Zn, might support the Gd-Zn transmetallation as mechanism of Gd retention.

### Significance and possible toxicity

Gadolinium belongs to the group of rare-earth elements, it is normally not found in living organisms, and it is highly toxic in its free ionic Gd^3+^ form^3^. Bone tissue is metabolically active, and continually undergoes remodelling. Therefore, slow endogenous Gd release into the bloodstream is likely to occur, and the risk is even higher in subjects with increased bone resorption (pregnancy, lactation, during menopause; in osteoporotic patients)^17,45^. Increase in safety concerns regarding the use of GBCAs triggered animal research, investigating Gd retention by various tissues, under single or repeated administration of GBCAs in healthy animals, as well as in induced disease models. Jost *et al*. compared linear and macrocyclic GBCAs with regard to brain deposition in rats after 2 weeks of repeated administration using LA-ICP-MS^46^. Previously unknown site of Gd accumulation was identified by Delfino *et al*., who observed Gd deposition in the periodontal tissues in murine model with induced renal disease, using SR-XRF and LA-ICP-MS^47^. Interesting results showing differential accumulation of Gd by different bone tissues – cortical, trabecular bone and bone marrow in juvenile and adult rats by ICP-MS were published by Fretellier *et al*.^48^. An interesting line of research – usage of GBCAs in pregnancy and potential effect of Gd on foetus, were investigated by Prola-Netto *et al*. in rhesus macaques, and although only extremely low levels of Gd were found in juvenile tissues after in utero exposure, femur was identified as a site of consistent Gd retention in all the animals^49^. Until now, however, the studies allowing elucidation of mechanisms of Gd incorporation into bone, as well as its further fate, were not undertaken, therefore, animal models and studies involving human biopsies are called for^50^.

The knowledge about deposition of Gd from GBCAs in bone is of importance, especially in the view of recently gained evidence of its accumulation behaviour in brain tissue, and possible risks associated with toxicity of free Gd. To the best of our knowledge these measurements are the first attempt of imaging Gd accumulations in the bone tissue, which is of exceptional value for understanding the mechanisms of Gd retention and, further, for predictions regarding the safety of GBCAs.

### Outlook

Summarizing the key questions for the further research we suggest to focus on: (i) systematic analysis of biopsies from patients with known history of GBCA intake in comparison with controls without clinical Gd exposure, (ii) animal studies comparing Gd supplemented and control animals to distinguish accumulation patterns for continuous, as well as short time exposure events, (iii) quantification of Gd within bone which can be achieved using matrix-matched standards (the other elemental imaging methods, such as LA-ICP-MS and SIMS could be applied as well); (iv) the speciation of the deposited Gd has to be performed, e.g. by XANES and EXAFS to gain knowledge about the specific chemical form, which is essential in predicting the possible health hazard (toxicity).

## Supporting information

Supporting information.
Supporting information2.
Supporting information3.
Supporting information4.
Supporting information5.
Supporting information6.
Supporting information7.
