# Supplementary figures and images for: Detection and imaging of gadolinium accumulation in human bone tissue by micro- and submicro-XRF
Source: Sci Rep. 2020 Apr 14;10:6301. doi: 10.1038/s41598-020-63325-9 (PMC7156386; doi:10.1038/s41598-020-63325-9)

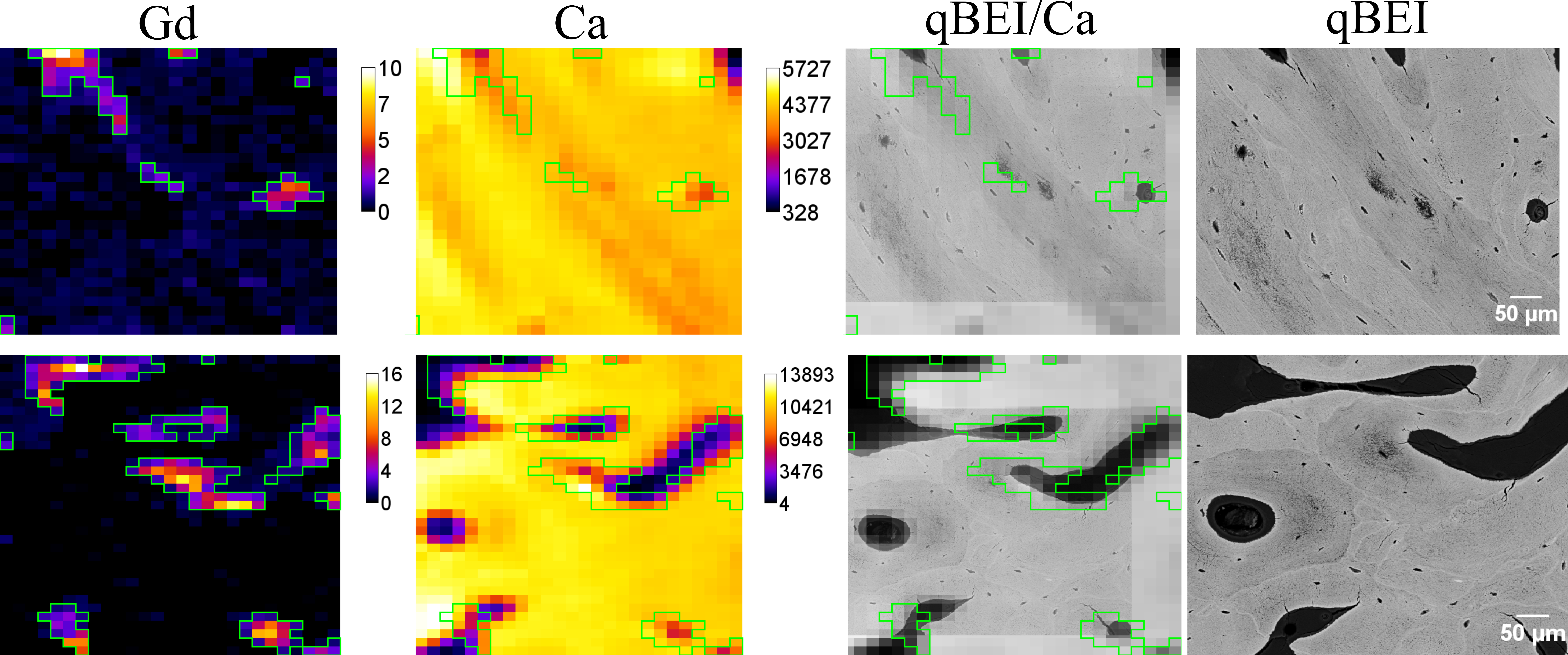

Supplement: Supplementary file 1 — Supporting information. [file 41598_2020_63325_MOESM1_ESM.png]

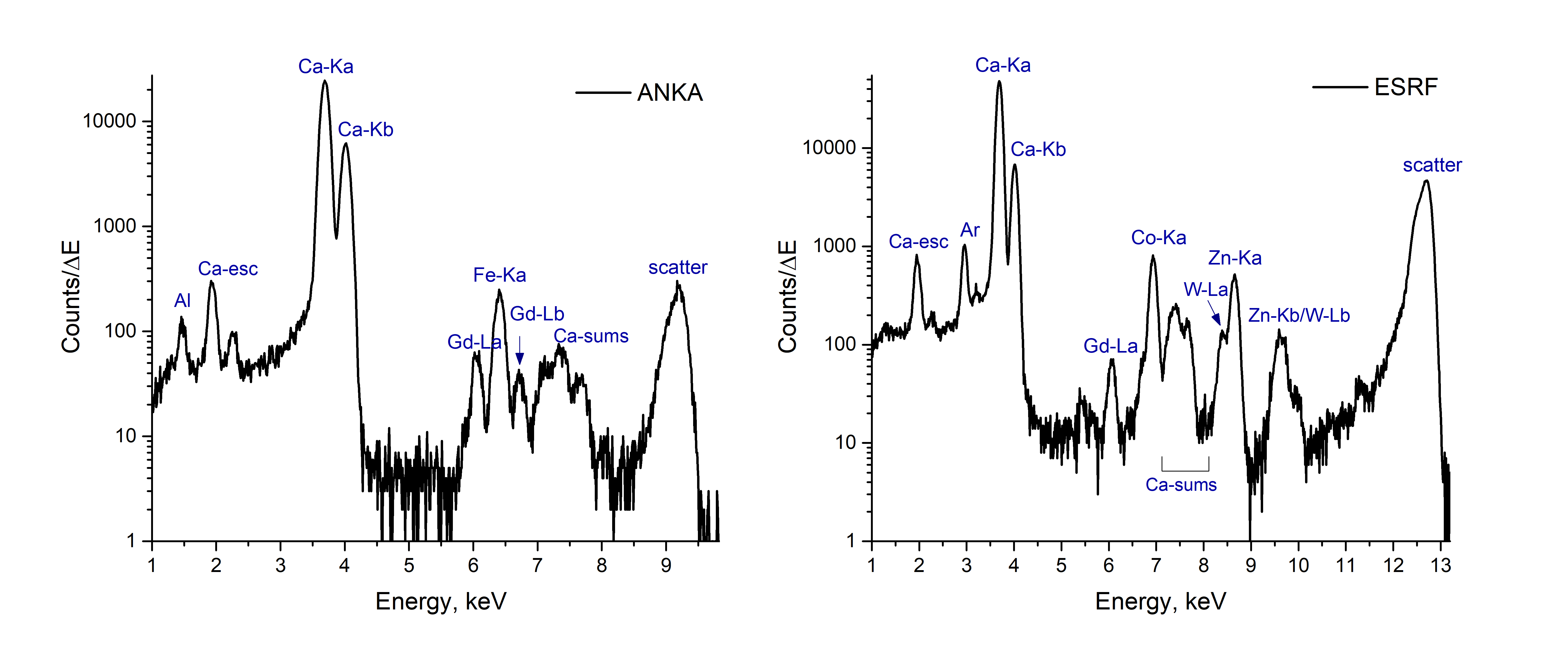

Supplement: Supplementary file 2 — Supporting information2. [file 41598_2020_63325_MOESM2_ESM.png]

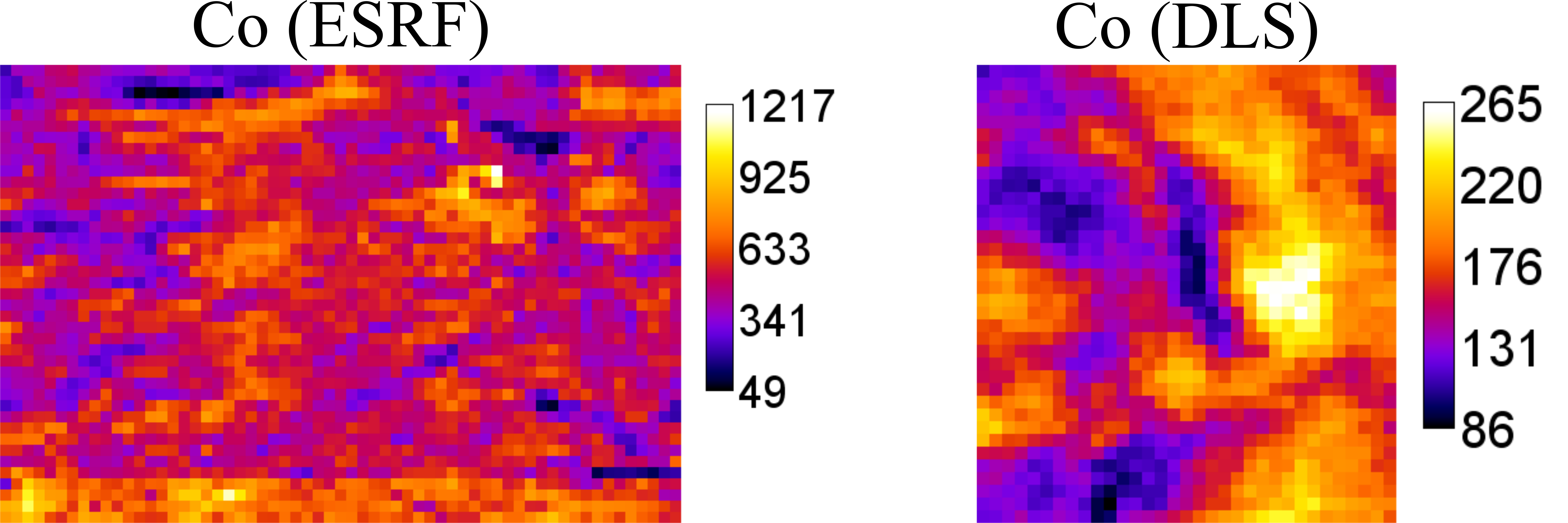

Supplement: Supplementary file 3 — Supporting information3. [file 41598_2020_63325_MOESM3_ESM.png]

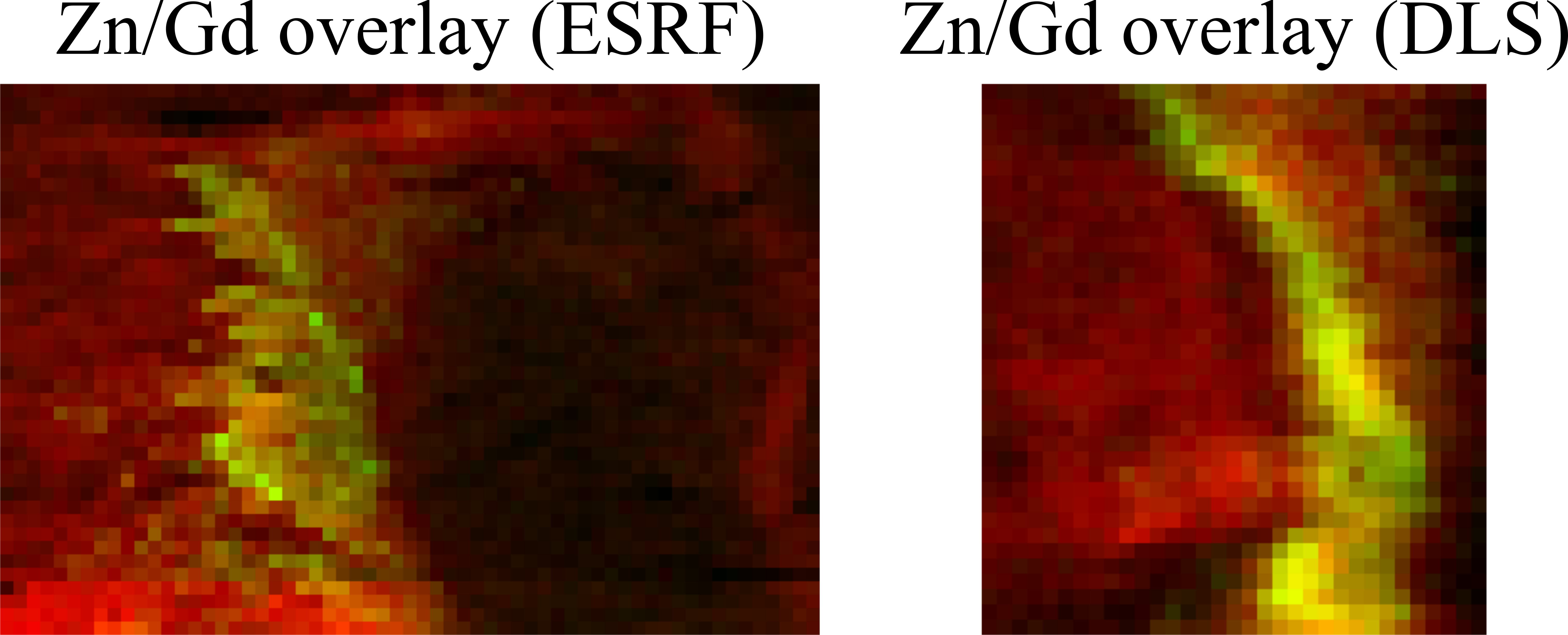

Supplement: Supplementary file 4 — Supporting information4. [file 41598_2020_63325_MOESM4_ESM.png]

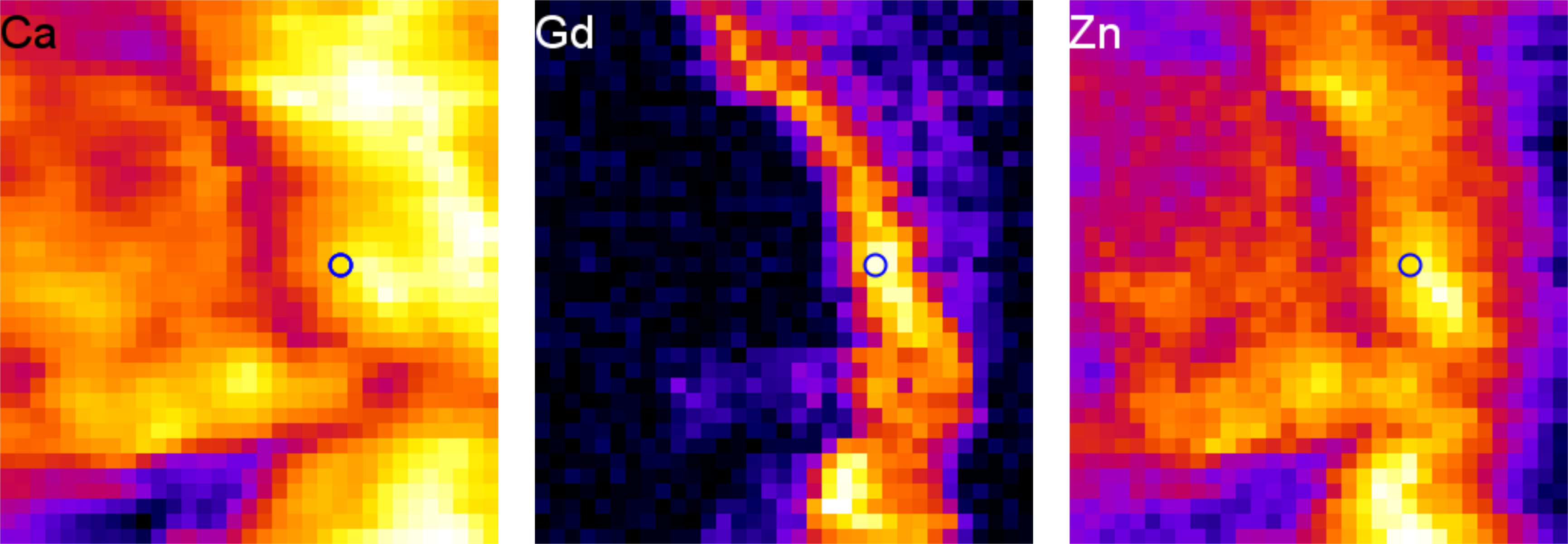

Supplement: Supplementary file 5 — Supporting information5. [file 41598_2020_63325_MOESM5_ESM.png]

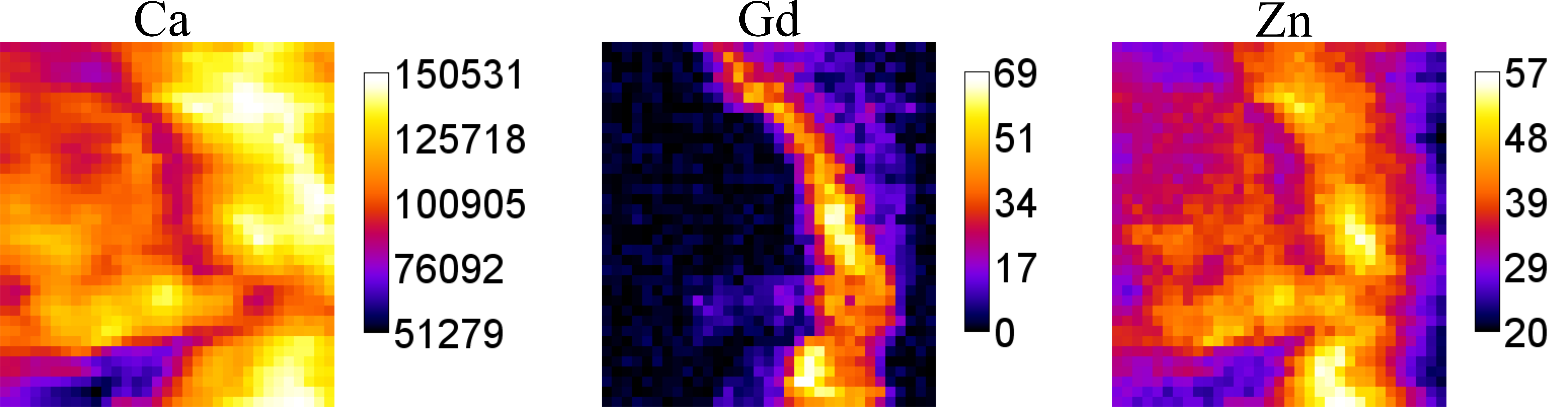

Supplement: Supplementary file 6 — Supporting information6. [file 41598_2020_63325_MOESM6_ESM.png]
