# Supplementary material for: Detection and imaging of gadolinium accumulation in human bone tissue by micro- and submicro-XRF
Source: Sci Rep. 2020 Apr 14;10:6301. doi: 10.1038/s41598-020-63325-9 (PMC7156386; doi:10.1038/s41598-020-63325-9)
Supplement: Supplementary file 7 — Supporting information7. [file 41598_2020_63325_MOESM7_ESM.docx]

**Detection and imaging of gadolinium accumulation in human bone tissue by micro- and submicro-XRF**

Anna Turyanskaya^1^*, Mirjam Rauwolf^1^, Vanessa Pichler^1^, Rolf Simon^2^, Manfred Burghammer^3^, Oliver J. L. Fox^4^, Kawal Sawhney^4^, Jochen G. Hofstaetter^5,6^, Andreas Roschger^5,7^, Paul Roschger^5^, Peter Wobrauschek^1^ and Christina Streli^1^

**Supplementary Material:**

The maps shown on Fig. S1 were obtained during second beamtime at ANKA. The description of the setup is provided in Materials and Methods section.

As the setup could have been adjusted differently, Gd signal is lower. Due to specifics of the spectra, the spectral background was fitted with the negative values for Gd. Therefore, in order to improve the contrast, negative values on these Gd maps were set to zero. In addition, in the Gd map of the region shown in the bottom row, was introduced another threshold – Cr-Mn-Fe-Ni contamination was identified in the hole, and this region was masked, and the values were also set to zero.


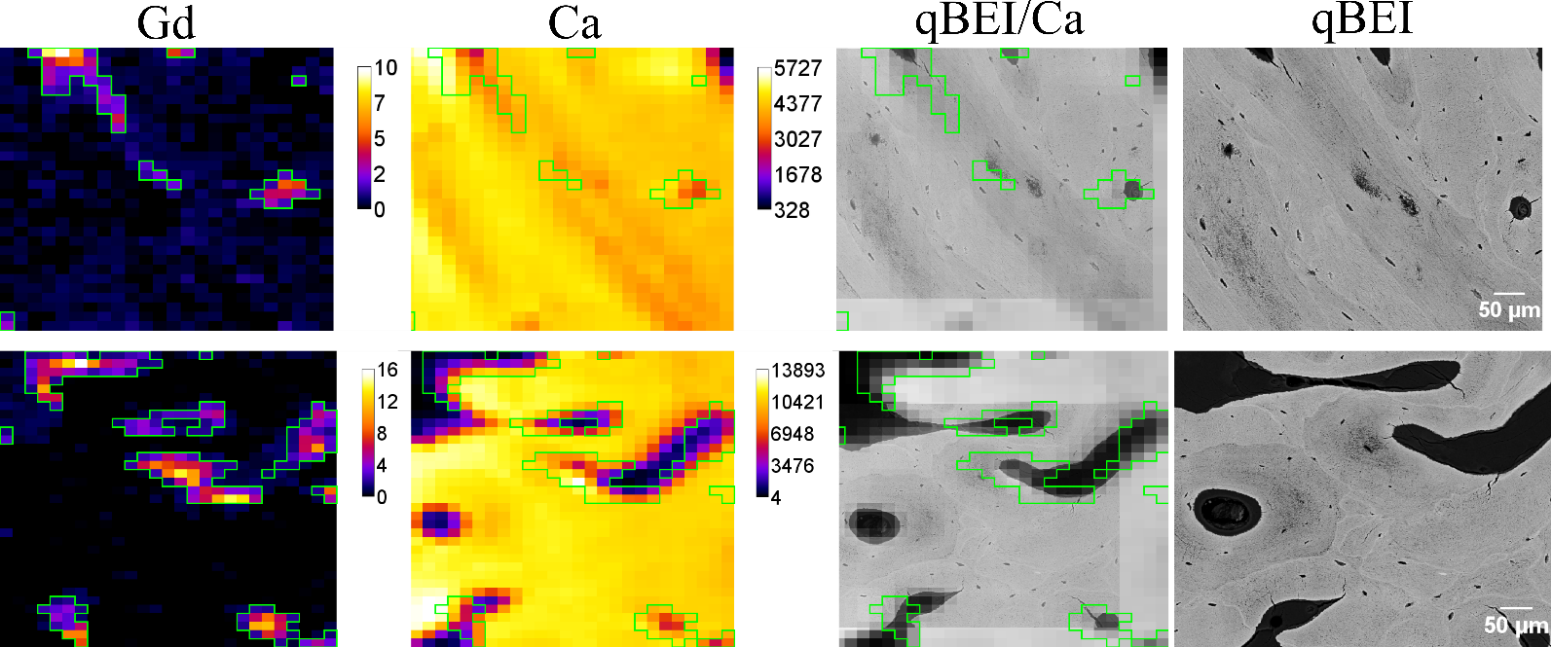


Figure S1. Gd and Ca elemental maps (in cps) obtained at ANKA, composite image and corresponding qBEIs of two areas.

Top image: area size for maps 24 x 30 pixels, 575 x 493 µm^2^;

Bottom image: area size maps 27 x 35 pixels, 650 x 578 µm^2^;

Dimensions for both qBEIs 570 x 450 µm^2^.


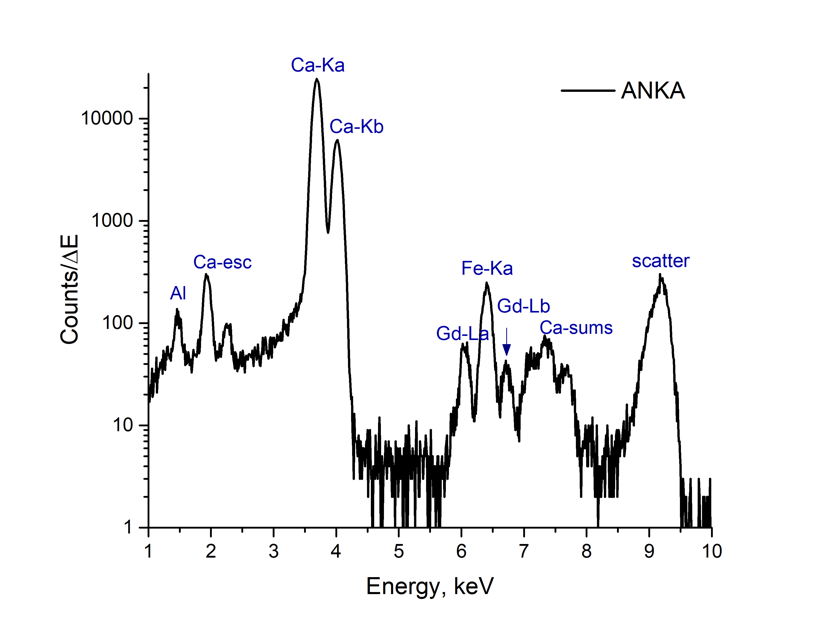

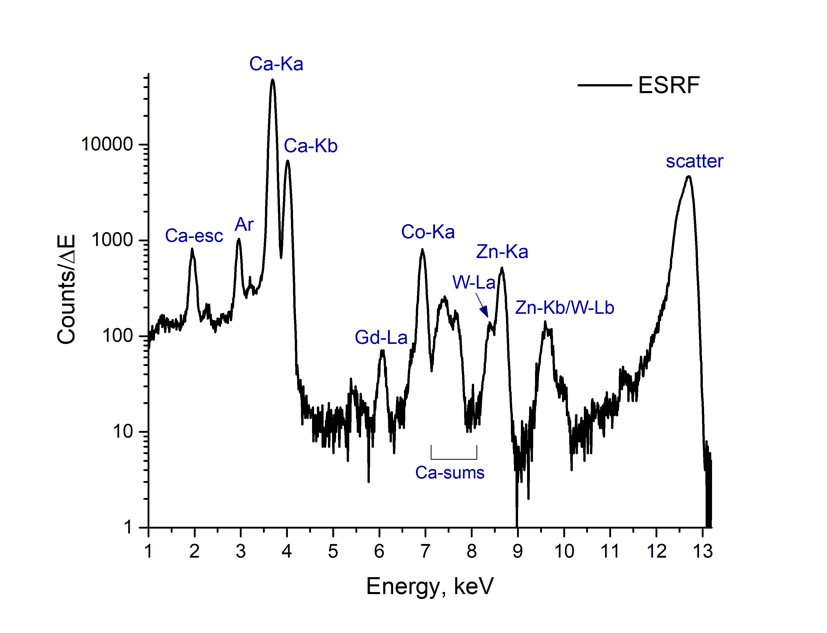


Figure S2. Exemplary spectra featuring gadolinium: ANKA (left) and ESRF (right).


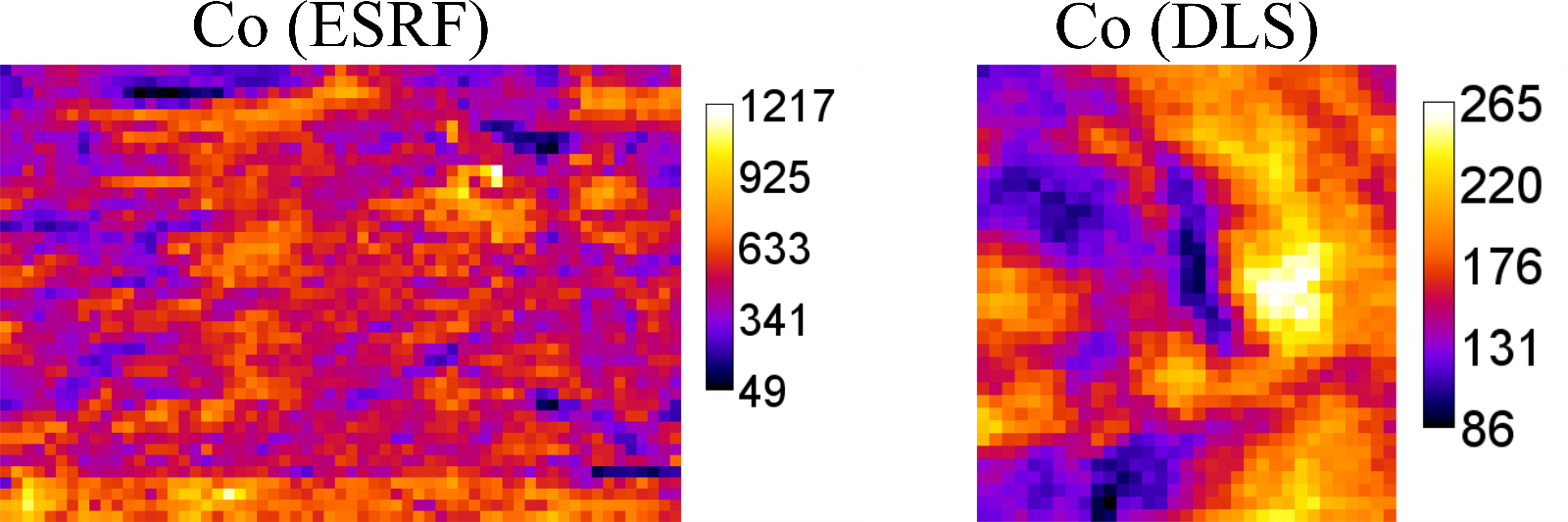


Figure S3. Co maps (in cps): ESRF (left, corresponds to Fig. 2); Diamond Light Source (right, corresponds to Fig. 3).


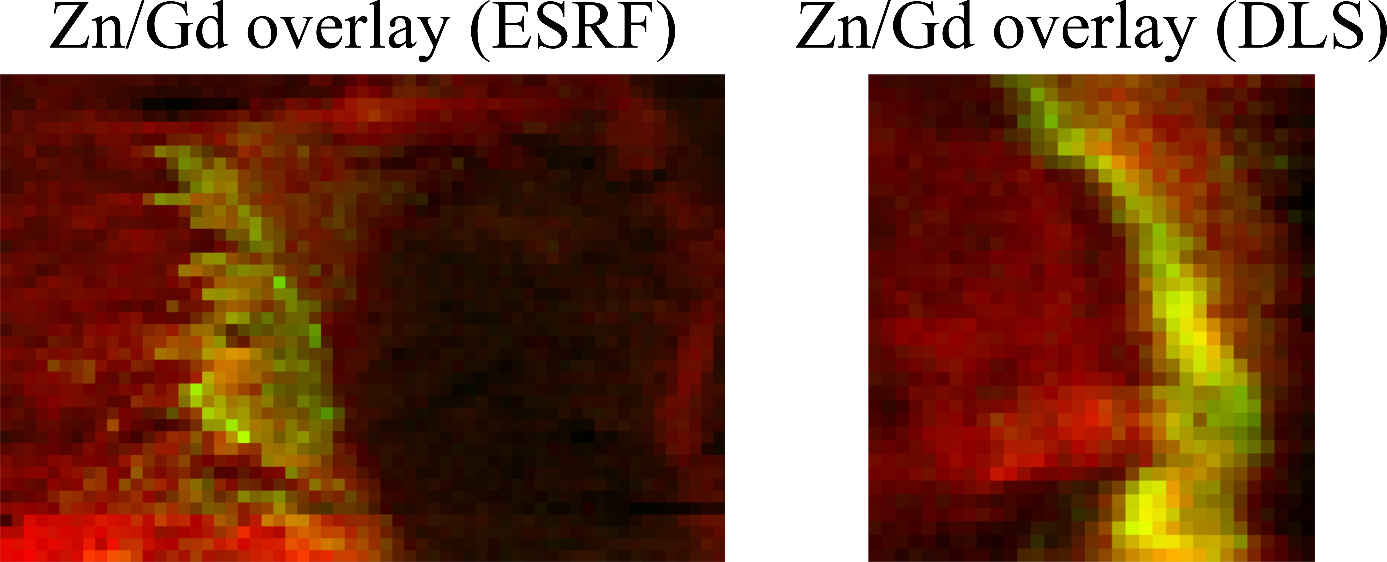


Figure S4. Composite images of Zn (red) and Gd (green) overlays from ESRF (left, corresponds to Fig. 2) and Diamond Light Source (right, corresponds to Fig. 3).

**Quantification attempt**

We performed quantification of the maps obtained at DLS with two different methods. We are confining the analysis to these maps due to the following reasons:

1. In thin sample absorption matrix effects can be neglected – and the sample measured at DLS is a thin cut;
2. During the same beamtime at DLS we measured reference material (further details below).

Further, since the section measured at ESRF and DLS was the same one, the obtained concentrations can be safely applied in relation to ESRF maps as well.

While the areas measured at the ESRF and DLS are different – and, therefore the concentrations can differ as well, the ratios, obtained using maximum values of Ca, Zn and Gd are well aligned (see the table 1 below).

*Table 1. Ratios of maximal values for Ca, Zn, Gd - from ESRF and DLS elemental maps (in cps)*

| **Element ratio** | **ESRF** | **Diamond Light Source** |
| --- | --- | --- |
| Ca:Gd | 47.7 | 43.8 |
| Ca:Zn | 248.6 | 247.1 |
| Zn:Gd | 5.2 | 5.6 |

**Quantification approach 1. Fundamental parameters**

Application of fundamental parameters method for quantification requires certain information to be known (generally it consists of two steps – calibration, using the sample of known composition and then analysis of unknown sample). In case of our samples, such information is not available. Further limitation is the intrinsic inhomogeneity of the sample. Therefore, we can perform the calculations for one pixel only.

Since bone Ca content is better explored, than other elements, we will use Ca content as a reference. We select one pixel from the DLS map – where all elements of interest are present – and for the calculations assume, that the value of Ca in this point is 100 000 µg/g – which is close to the values reported by (Ščančar et al. 2000) for the same kind of material – transiliac bone samples.

The results for calculated concentrations for Gd and Zn are given in table 2.

*Table 2. FP-based content of elements in point 22:17 of DLS map*

| **Element** | **Concentration, µg/g** | **Value in cps** |
| --- | --- | --- |
| Gd | 267.5 | 45 |
| Zn | 197.4 | 238 |
| Ca | 100 000 (putative) | 10375 |

The point with coordinates 22:17 (x:y) is marked on the Fig. S5 below:


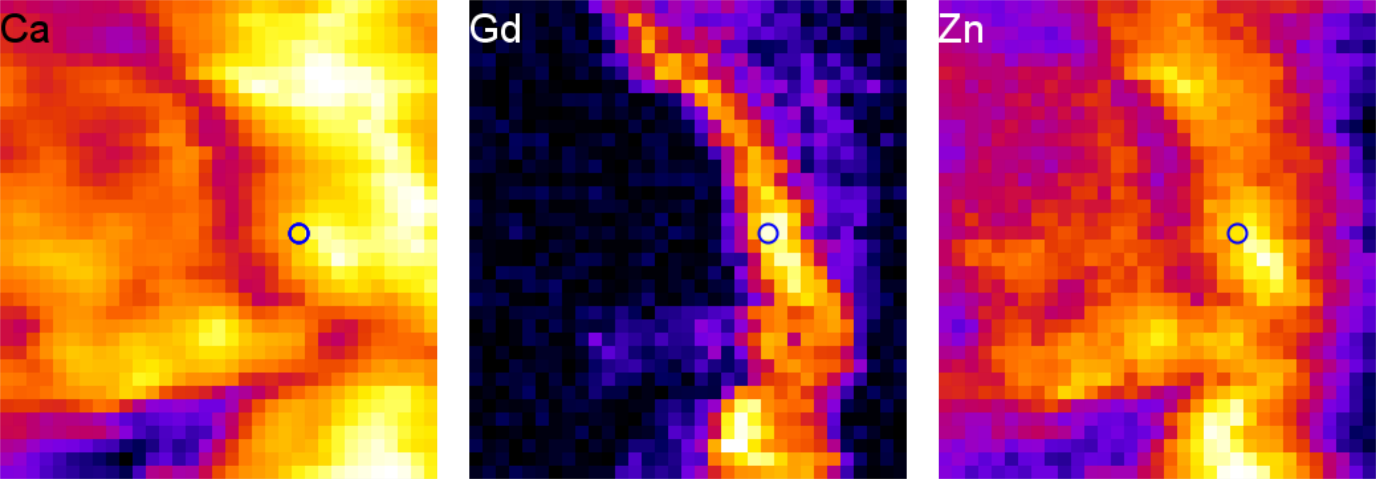


Figure S5. Ca, Gd and Zn maps obtained at DLS with the pixel considered for FP method approach – marked with circle

However high the obtained value for Gd might seem, it will only increase, if we plug a greater number for Ca concentration into the equation – although, one also should bear in mind, that this is only the single-pixel value within the accumulation sight, and therefore cannot be really compared with the averaged bulk values given in literature.

**Quantification approach 2. Recalculation with sensitivities estimated from standard reference material**

During the same beamtime we also measured standard reference material, NIST SRM 1412 – Multicomponent glass, in order to characterize the setup. The experiment and procedure are described in (Rauwolf et al. 2018). The glass is a fitting reference material for our purposes, since it contains the elements, which can be exited with 12.7 keV via L-lines. The sensitivities for Zn and Ca could be taken directly from the standard measurements – and the sensitivity for Gd had to be extrapolated based on the known values for Sr, Cd and Ba; the values are included in table 3, and the maps recalculated in µg/units are presented below. But the glass standard is not a thin film standard, so the obtained sensitivities are supposed to be lower than for thin film approximation leading to lower concentration values of Ca, Gd and Zn for the bone sample.

*Table 3. Sensitivities K/L-lines - SRM NIST 1412*

|  | **Element** | **Sensitivity, cps*g/µg** | **Comment** |
| --- | --- | --- | --- |
| K | Ca | 0.079 |  |
|  | Zn | 4.7 |  |
| L | Gd | 0.7 | extrapolated value |


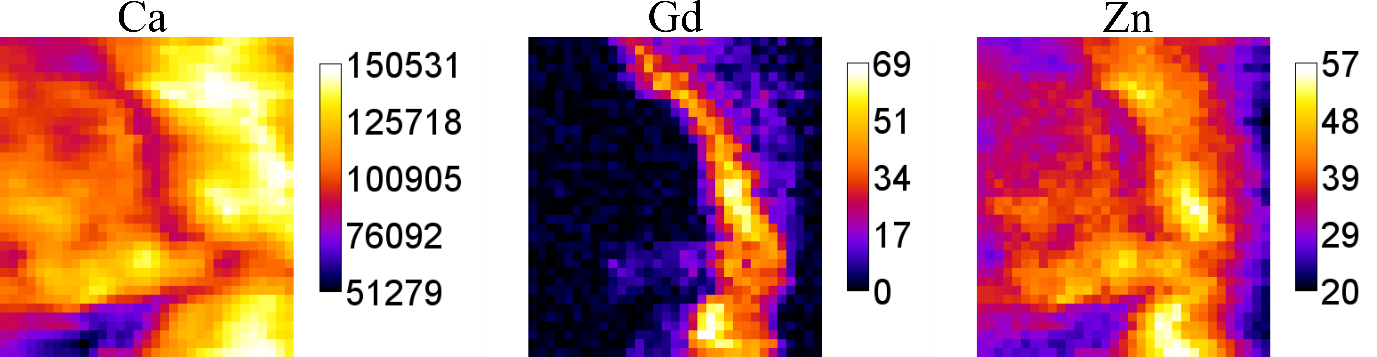


Figure S6. Ca, Gd and Zn elemental maps (in µg/g) from Diamond Light Source

As Ca content is the best “reference” to judge about the obtained result – the range 50 000-150 000 µg/g (seen from the calibration bars of the Fig. S6) seems reasonable according to literature values (Ščančar et al. 2000; Zioła-Frankowska et al. 2015). With all the given limitations, the Zn values also do not fall far from the range given by Zioła-Frankowska et al. – ca. 40-110 µg/g.

**Conclusion**

The obtained Gd concentration can only be given as indicative, and, since both used methods of quantification have certain limitations, it might be more correct to provide the range for the maximum content, i.e. 70-270 µg/g. This value obtained from the small bone area and representing localized Gd accumulation by no means can be compared with the literature averaged bulk values.

***References:***

Rauwolf, M., A. Turyanskaya, D. Ingerle, N. Szoboszlai, I. Pape, A. W. Malandain, O. J. L. Fox, L. Hahn, K. J. S. Sawhney, and C. Streli. 2018. “Characterization of a Submicro-X-Ray Fluorescence Setup on the B16 Beamline at Diamond Light Source.” *Journal of Synchrotron Radiation* 25(4):1189–95.

Ščančar, Janez, Radmila Milačič, Miha Benedik, and Peter Bukovec. 2000. “Determination of Trace Elements and Calcium in Bone of the Human Iliac Crest by Atomic Absorption Spectrometry.” *Clinica Chimica Acta* 293(1–2):187–97.

Zioła-Frankowska, Anetta, Łukasz Kubaszewski, Mikołaj Dąbrowski, Artur Kowalski, Piotr Rogala, Wojciech Strzyżewski, Wojciech Łabędź, Ryszard Uklejewski, Karel Novotny, Viktor Kanicky, and Marcin Frankowski. 2015. “The Content of the 14 Metals in Cancellous and Cortical Bone of the Hip Joint Affected by Osteoarthritis.” *BioMed Research International* 2015:1–23.
